# Implementing a comprehensive newborn monitoring chart: Barriers, enablers, and opportunities

**DOI:** 10.1371/journal.pgph.0000624

**Published:** 2022-07-25

**Authors:** Naomi Muinga, Ibukun-Oluwa Omolade Abejirinde, Lenka Benova, Chris Paton, Mike English, Marjolein Zweekhorst

**Affiliations:** 1 Athena Institute, VU University Amsterdam, Amsterdam, Netherlands; 2 KEMRI/Wellcome Trust Research Programme, Nairobi, Kenya; 3 Department of Public Health, Institute of Tropical Medicine, Sexual and Reproductive Health Group, Antwerp, Belgium; 4 Dalla Lana School of Public Health, University of Toronto, Toronto, Canada; 5 Women’s College Hospital Institute for Health System Solutions and Virtual Care, Toronto, Canada; 6 Nuffield Department of Medicine, Centre for Tropical Medicine and Global Health, University of Oxford, Oxford, GB, England; 7 Department of Information Science, University of Otago, Dunedin, New Zealand; Brigham and Women’s Hospital, GUATEMALA

## Abstract

Documenting inpatient care is largely paper-based and it facilitates team communication and future care planning. However, studies show that nursing documentation remains suboptimal especially for newborns, necessitating introduction of standardised paper-based charts. We report on a process of implementing a comprehensive newborn monitoring chart and the perceptions of health workers in a network of hospitals in Kenya. The chart was launched virtually in July 2020 followed by learning meetings with nurses and the research team. This is a qualitative study involving document review, individual in-depth interviews with nurses and paediatricians and a focus group discussion with data clerks. The chart was co-designed by the research team and hospital staff then implemented using a trainer of trainers’ model where the nurses-in-charge were trained on how to use the chart and they in turn trained their staff. Training at the hospital was delivered by the nurse-in-charge and/or paediatrician through a combined training with all staff or one-on-one training. The chart was well received with health workers reporting reduced writing, consolidated information, and improved communication as benefits. Implementation was facilitated by individual and team factors, complementary projects, and the removal of old charts. However, challenges arose related to the staff and work environment, inadequate supply of charts, alternative places to document, and inadequate equipment. The participants suggested that future implementation should be accompanied by mentorship or close follow-up, peer experience sharing, training at the hospital and in pre-service institutions and wider stakeholder engagement. Findings show that there are opportunities to improve the implementation process by clarifying roles relating to the filing system, improving the chart supply process, staff induction and specifying a newborn patient file. The chart did not meet the need for supporting documentation of long stay patients presenting an opportunity to explore digital solutions that might provide more flexibility and features.

## Introduction

Documenting care is an important step in ensuring good outcomes for patients, particularly newborns, who may be admitted for extended periods and receive care by multi-disciplinary teams. Documentation facilitates intra- and interdisciplinary team communication and future care planning [1]. Despite the move toward digital systems, paper-based medical records continue to dominate the inpatient setting in both low- and middle-income settings and some high-income settings [2–4] and there have been efforts to improve documentation by implementing better paper-based monitoring charts [4, 5].

Studies on improving documentation of care are embedded in quality improvement projects or health systems research, which involves implementing an innovation that introduces a new way of working or a new product that benefits the unit of adoption [6]. However, few quality improvement initiatives are sustained beyond the pilot and initial implementation stages [7] and evidence on the routinization of innovations is sparse [8]. Therefore, there is a need to generate evidence on how the implementation of innovations happen, to inform decisions on the implementation process and promote collective learning.

Studies have shown that the quality and quantity of nursing documentation needs improving to support patient care [9, 10]. In Kenya, documentation of inpatient paediatric and newborn care has been suboptimal, limiting the use of routine data for quality improvement [11, 12]. This has led to initiatives to improve the documentation of inpatient newborn care by introducing standardised paper-based charts such as the comprehensive newborn monitoring chart that was developed using a Human-centred Design (HCD) approach [13].

This study builds on the earlier work of chart development and piloting [13] by examining the process of implementing the comprehensive newborn monitoring chart and the perceptions of health workers. It also identifies opportunities for improving the implementation process and contributes to the literature by generating evidence on the implementation process.

## Methods

### Ethics statement

Ethical approval for this study was granted by the Kenya Medical Research Institute (KEMRI) Scientific and Ethics Review Unit (Protocol no: KEMRI/SERU/CGMRC/161/3852). Verbal or written consent was obtained from participants. The study information sheet was sent via email and at the start of the telephone interviews, the researcher gave a summary of the study to ensure the participants understood what the study is about. The interview participants were not reimbursed, but the data clerks participating in the FGD were reimbursed with 1 GB worth of data since the discussion took place online via Zoom software. Additional information regarding the ethical, cultural, and scientific considerations specific to inclusivity in global research is included in the supporting information (S3 Text).

### The comprehensive newborn monitoring chart

The chart contains 5 distinct sections: biodata, feed and fluid prescription, vitals and assessment monitoring, nursing shift notes and an input (feed and fluid) balancing section (S1 Text). It is designed to cover 48 hours if printed double-sided on A4 paper. A large section of the chart contains 12 columns for documenting repeated measures, it can be used flexibly as there are no fixed timings in the top row. This design was developed to maximise the use of paper in a low resource setting while allowing for comprehensive monitoring of vital signs, feed, and fluid documentation on a single sheet. All sections are filled by the nursing team except the prescription section, which is filled by the clinicians. Along with the chart, we co-developed an information sheet that provides normal and out-of-range values for vital signs (temperature, respiratory rate, pulse, oxygen saturation, and blood sugar) and identification of respiratory distress (S2 Text) to support health workers in identifying patient deterioration.

The chart was developed through a series of co-design workshops facilitated by the research team (NM, ME and CP) and therefore there existed a prior relationship with some of the interviewees.

### Design

This is a qualitative study involving document review, individual in-depth interviews (IDIs) and a focus group discussion (FGD). We follow the consolidated criteria for reporting qualitative research (COREQ) guidelines for this report [14].

### Setting

This study leverages an established Clinical Information Network for Newborns (CIN-N) comprising 20 county referral hospitals located in Nairobi, the Central, Eastern, and Western parts of Kenya. CIN-N is a partnership between researchers, the Kenyan Ministry of Health, the Kenya Paediatric Association, and county hospitals that provides a database of patient-level data from all newborn admissions at discharge [15, 16].

Recent data from the network shows that the crude mortality rate stands at 10.2% (95% CI 9.97% to 10.55%) [17]. The newborn unit staff comprises 1–2 paediatricians (or a neonatologist) per hospital and 7–21 nurses (administrative and clinical). Previous work has reported a median ratio of 1 nurse to 19 babies and a maximum exceeding 25 babies in public hospitals [18]. Other staff may include student nurses, medical officers (clinically qualified but no specialization), medical officer interns, clinical officers (non-physician clinicians), clinical officer interns, and nutritionists. Concerning the chart, the clinicians (paediatricians, medical officers, and clinical officers) usually write a feed or fluid prescription, while nurses repeatedly document observations on babies’ condition during admission (inpatient monitoring). The central Records Department in all hospitals is staffed by health records information officers who are responsible for the procurement and supply of medical records, among other tasks. In the CIN-N, the data clerks abstract data from patient files and enter it into an electronic database.

Successful implementation of the chart will contribute to better tracking and monitoring of inpatient newborn care within and across hospitals in the CIN-N with the potential to scale up to more hospitals across Kenya and other similar settings. Successful implementation would also support two further initiatives, the Newborn Essential Services and Technologies (NEST) Program and a newborn clinical audit project. The NEST program collaborates with governments to strengthen health systems in Nigeria, Tanzania, Malawi and Kenya through innovative technology, education, and policy resources [19]. The program currently runs in 13 hospitals in the CIN-N. The clinical audit project is promoting reviews of the quality of care provided to newborns in four CIN-N hospitals as part of local improvement efforts.

### Implementation strategy of the comprehensive newborn monitoring chart

**Fig 1:**
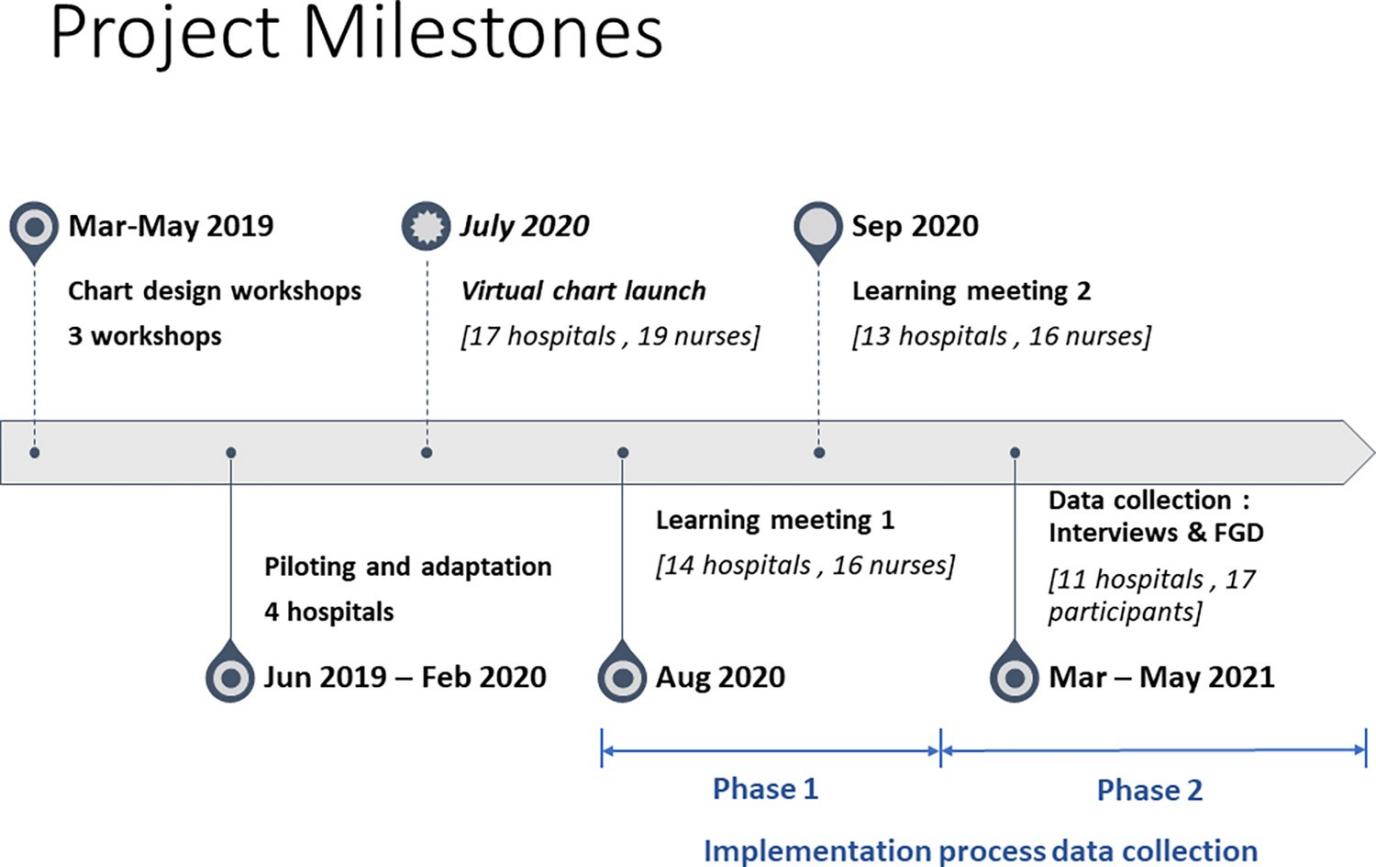
Project timeline.

The initial face-to-face meeting chart launch slated for April 2020 was changed to a virtual launch in July 2020 after discussion with hospital-based nurses and the research team following physical meeting restrictions at the onset of the COVID-19 pandemic. We conducted two training and consensus building meetings via Zoom facilitated by the research team (NM and EG) and attended by nurses-in-charge who manage the newborn wards: 19 participants representing 17 hospitals in the CIN-N network. Some invited network members were unable to join the meetings because of limited time or unfamiliarity with technology, which was an initial barrier to online meeting participation. The meetings allowed us to get consensus on the final design of the monitoring chart (urine assessment revised to a Yes/No field from diaper count), review each section and discuss how to fill it. These meetings, therefore, served as trainer of trainers training sessions with nurses who were expected to become champions of chart implementation and use. Following this, we facilitated two learning meetings attended by Nurses-in-charge of 14 newborn units in August and September 2020 to discuss emerging issues immediately following chart launch. The learning meetings were conducted as part of the implementation process and quality improvement initiative by NM & EG, and they served as a platform for experience sharing among participants. All newborn nurses-in-charge in the network were invited to participate. **Fig 1** shows the distribution of participants, hospitals represented, and timeline of activities from the chart design phase to implementation.

A distribution package was sent to the nurses-in-charge of the newborn units in August 2020 containing a two-month supply of the comprehensive newborn monitoring charts, sample-filled charts, a colour version of the information sheet to be used as a wall poster in the newborn unit, a training sheet describing all fields on the chart and a PowerPoint slide deck to be used by the nurse-in-charge to train the staff. We adopted this training of trainers’ model because the team felt that it was the best model at the time, and it would also encourage the uptake of charts among the nurse-in-charges. While the nurses were free to introduce the charts as they saw fit, we encouraged them to conduct a training session with all staff (nurses, clinicians, and nutritionists) at the newborn units on how to use the monitoring charts. We also trained the data clerks who are part of the data collection team in the CIN-N, on the new monitoring chart during the research team’s data quality meetings.

### Data collection

Multiple data sources were used to understand the chart implementation process over two phases as illustrated in **Fig 1**. Phase one included meeting notes from 2 online learning meetings conducted 2 months after the virtual launch (August and September 2020). Phase two included telephone-based IDIs with nurses and paediatricians responsible for managing the newborn ward and an FGD with data clerks via Zoom online platform conducted 7 months after the virtual chart launch (March-May 2021). Throughout the implementation period, NM maintained project implementation notes that included meeting notes, personal reflections, field notes, ad hoc conversations with the nurses-in-charge and research team debrief sessions that occurred via telephone. An online approach was preferred due to the physical meeting restrictions that were in place at the onset of the COVID19 pandemic.

The meeting notes from phase 1 learning meetings were used to inform the development of questions that were included in the subsequent in-depth interviews in phase 2, thereby providing an opportunity to explore emerging and persisting issues with the implementation. The online learning meetings adopted an open format to encourage the participants to express their views on the implementation process.

The participants were purposively selected to represent those who had a role in implementing the charts. A total of 34 nurses and paediatricians were invited to participate in the interviews via email that was followed up by a text message and a phone call. Through a snowballing process, we asked the participants to suggest other potential interviewees, who could provide relevant information on the topic of discussion. Interview questions explored participants’ perceptions of the chart, implementation experience, perceived benefits, challenges, and opportunities for improving future initiatives. The interview guide was designed to follow up on issues that emerged during the learning meetings, and it was adapted throughout the interviews. Subsequent interviews sought to explore issues emerging from initial interviews to assess whether the phenomena reported in one facility were experienced in other facilities or to understand a phenomenon better through different interviewees from the same hospital. The interviews lasted between 30 and 60 minutes depending on participant availability and their knowledge of the implementation process.

The FGD that lasted 2 hours was conducted midway through the IDIs and aimed to explore issues concerning: the type of patient file, supply of monitoring charts to the newborn ward, and completeness and quality of entry. FGD participants were recruited purposively from six hospitals that have either a bound booklet type file where all patient charts are bound together or a loose-leaf type where a folder is used as a cover and charts are punched and attached by a string.

NM conducted the interviews while NM and PM conducted the FGD between March and May 2021. Audio recordings were transcribed verbatim by a professional transcription team and proofread by NM.

### Data analysis and triangulation

Two researchers (NM & PM) coded two IDI transcripts and developed an initial codebook that was revised to develop a midway codebook following discussion between NM, IOA and MZ. After further review, a final codebook was applied to the remaining transcripts. Codes were developed inductively and clustered into themes, grouping related codes through an iterative process. Additionally, the data synthesis incorporated research team conversations and debriefings to understand themes and make linkages between them. Overall, one researcher (NM) read and coded all the transcripts while three researchers (NM, PM, IOA) coded three transcripts to ensure consistency of codes. Data analysis was supported using NVIVO 12 Pro: QSR International Pty Ltd, 2019. We applied triangulation by comparing which issues arose during the two phases of data collection and between participants.

## Results

We conducted two learning meetings with nurses in phase one followed by interviews and an FGD 2 and 8 months after chart launch in phase two of data collection. The interviews included 12 participants (5 Nurse-in-charges, 2 nurses, 4 paediatricians, and 1 NEST coordinator) six of whom were involved in the chart design workshops, while the FGD involved five data clerks from five hospitals. Each participant was interviewed once. Overall, 12 hospitals in the CIN-N were represented in the second phase of data collection and 3 hospitals had two participants each (Table 1). Table 2 shows hospital characteristics and a snapshot of the number of babies and the number of nurses in a shift, as an indication of the workload as of June 2021, as well as a distribution of interviewees and FGD participants. The implementation period was characterized by periodic ward closures whenever there were COVID19 cases detected and there were no students as the training institutions were closed. Three of the hospitals used bound booklets, while the rest used loose-leaf files. Eight of the hospitals were in the NEST program, while five were part of the clinical audit study. Two hospitals(H3 –July 2019 and H11- Feb 2020) were early chart adopters as they participated in the pilot phase and continued to use the new monitoring chart after the piloting period [13].

**Table 1:** Overview of data collection.

Activity (date)	Total participants	Hospitals represented	Participants		
			Nurses	Paediatricians	Data clerks
Online launch (two meetings in July 2020)	19	17	X		
Learning meeting 1 Aug 2020	16	14	X		
Learning meeting 2 September 2020	16	13	X		
IDI and FGD data collection (12 hospitals)					
In-depth Interviews	12	10	X	X	
FGD	5	5			X

**Table 2:** Hospital characteristics.

Hospital code	Filetype as of Feb 2020 L = Loose-leaf B = Bound booklet	Staffing as of June 2021		Complementary projects		Data collection IDI and/or FGD
		No of newborns in the Ward	Number of nurses in a shift	NEST	Clinical audit study	
H1	L	40	3	Y		IDI + FGD
H2	B	25	1–2	Y		IDI
H3*	L	20	2	Y		IDI + FGD
H4	B	39	2–3	Y		IDI
H5	L	80	5	Y	Y	IDI
H6	L	91	13	N	Y	IDI
H8	L	40	1	Y		IDI
H9	B	66	2–4	Y	Y	IDI + FGD
H10	L	37	1–2	N	Y	IDI
H11*	L	65	3–4	Y	Y	FGD
H12	L	60	3	N		FGD

*Early adopter hospitals

**Fig 2:**
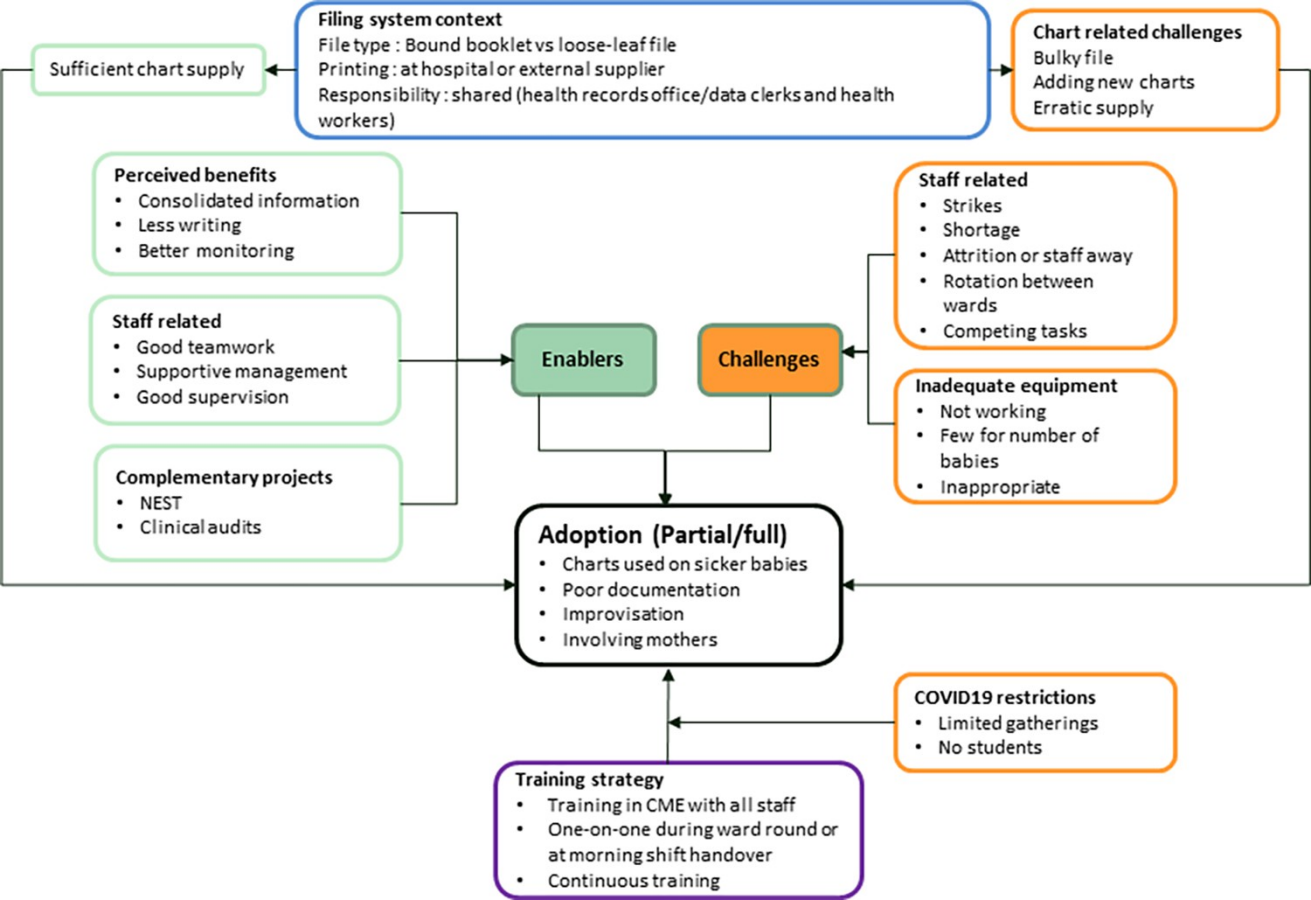
Summary of enablers, challenges and context leading to chart adoption.

We describe the chart implementation process and initial perceptions, filing system context, enablers of implementation, perceived benefits, challenges, and workarounds. The challenges, benefits and contextual issues led to the partial or full adoption of the monitoring charts as illustrated in **Fig 2**. Partial or full adoption was assessed by asking the respondents to describe how different sections of the chart were filled. Full adoption, therefore, refers to whether users documented on all sections of the chart based on the participants’ response. We also draw lessons for future implementations based on the participants’ suggestions and the supporting data. A codebook with coding frequencies has been provided as a S1 Data.

### Implementation

#### Implementation and initial perceptions of the chart

Following the online launch of charts, the newborn ward nurses-in-charge who served as focal persons were expected to train staff on the use of the monitoring chart. In some hospitals, the nurse worked with the paediatrician to deliver this training to all ward staff during a Continuous Medical Education (CME) session and followed up with one-on-one reminders to staff. In other hospitals, nurses-in-charge opted to introduce the charts through one-on-one sessions during daily ward rounds or during morning shift handover sessions. These one-on-one sessions were reportedly suitable due to COVID-19 restrictions limiting room capacities for in-person meetings. The one-on-one sessions were also preferred because staff work in shifts and often rotate between the newborn and paediatric wards, while group training only addressed those present at a given time.

Data from learning meetings and interviews showed that paediatricians and nurses-in-charge often supplemented the initial training sessions by conducting refresher sessions and reminding staff to use the charts. Analysis showed that while the training predominantly targeted nurses, the clinicians were also trained. Perhaps because the nurses were primarily involved in the design and a large portion of the chart is used to document nursing care. However, there was one exception in which the nurse-in-charge and paediatrician at H1 conducted a CME where all staff were present; both the paediatrician and nurse-in-charge at this hospital had been involved in the chart design phase.

“*I think it’s me who took the lead because we called a meeting and we introduced them to the charts*. *I called a meeting*, *the nurses in my department*, *then I introduced them to the charts*. *Then we had a CME with Dr XX to involve the doctors… so that we involve the doctors to show them that we are going to use those charts and how we expect them to be filling them”*. *IDI 01 Nurse-in-charge H1*

Initial perceptions from the two learning meetings and later with the interview respondents indicated that the hospital staff felt overwhelmed by the chart at the beginning of the implementation, because of the large amount of information required to complete it as illustrated in the quote below. The chart by design brought multiple sources of information together that was previously filled in separate documents.

“*I just think it is*, *they were seeing that there are so many places to be written*, *you know some people feel like*.. */so in my view*, *people feel like if you get something you just write one thing and you are through but this one keeps you down you have to be keen a bit for you to fill everything*.*” IDI 01 Nurse-in-charge H1*

However, interview data from phase 2 data collection shows that the staff later found it easier to use and it became accepted as instrumental in facilitating patient handovers, clinical audits, clinical training for the NEST program and general team communication as shown in the quote below.

“*now even in the morning when you’re taking report because we nurses*, *we hand over with the file so you read from the comprehensive chart*, *it’s easy to note in the morning what was not done and what was missed and the team are able to rectify rather than just giving*, *you know*, *handing over with everything like us we hand over with the file*. *So you’ll use that comprehensive chart to hand over” IDI 04 Nurse-in-charge H4*

#### Practical use of charts

The nurses in the learning meetings reported giving more attention to sicker babies and subsequently used the chart on this cohort in what appeared to be a phased implementation approach in the ward with sicker babies having well-filled charts. During the learning meetings, it emerged that documenting the input deficit was a challenge, with some nurses explaining that fluid balancing was rarely documented or documented in the nursing Kardex (unstructured nursing notes) as opposed to the monitoring chart. Similarly, the interviews conducted seven months later showed that the challenge persisted. Most interview respondents reported that the monitoring section that captures the vital signs was well filled while the feed and fluid prescription and the fluid balancing sections were not well filled. For feed and fluid balancing, the section at the bottom of the sheet allows the nurse to document how much input has been given at the end of each shift and how much is leftover. Any left-over amount should be carried forward so that the baby receives the total amount prescribed in 24hours. The nurses cited a lack of syringe pumps, other competing tasks and a high workload that left little time for detailed documentation at the point of care or end of shift documentation, as is shown by the following quote. The lack of appropriate equipment was further confirmed in the FGD discussion with data clerks and is elaborated in the challenges and workarounds section.

“*Just imagine every three hours, going to calculate the mls [millilitres] I have given and the drips remaining, ml’s remaining. I’m supposed to write a Kardex [nursing notes], I’m supposed to, to, do many things, it’s not possible…it is not." IDI 10 Nurse-in-charge H9(66 babies / 2 to 4 nurses per shift Table 2)*

During the learning meetings, it emerged that the clinicians preferred to document feed and fluid prescriptions in their notes or the treatment chart in keeping with their previous practice rather than the monitoring chart and later from the interviews, the feed and fluid prescription section was not being filled as expected. Other reasons for not filling the prescription section included: different clinician and nursing team reporting hours, staff rotation and shortage and limited supervision as illustrated in the quote by a paediatrician H2.

“*…because we’ve been leaving it [filling of the prescription] to the MOs [medical officers]*, *most of the time*, *but now we’re trying to make it more consistent in terms of a consultant being there and alternating and stuff*. *So in terms of filling it in*, *-from the medical point of view*, *it’s still not up to par*. *Yeah*, *-so we’re not*, *I don’t think we’ve done a good job in terms of emphasizing that which needs to be filled in and our part in it*.*” IDI 08 Paediatrician H2*

To overcome these challenges, the nurses-in-charge and paediatricians reported that they would remind the medical officers to document the prescription on the comprehensive monitoring chart. Interview data from two hospitals whose paediatricians had been involved in the design phase suggests that the clinicians may have received refresher training by the paediatricians as illustrated in the quote by the nurse-in-charge below. Generally, interviewees were optimistic that the documentation would improve over time.

“*Initially the nurses embraced it*, *the clinicians lagged behind like they still want to stick to their clerking notes but our paediatrician*, *she was also involved in the process of making it so she understood and she has been able to bring them on board but the nurses had no problem*. *Actually*, *it was a blessing to them” IDI 004 Nurse-in-charge H4*

### Filing system context and roles

The type of file system at the hospital (bound booklet or loose-leaf file) determined how quickly hospitals could incorporate new charts into medical records. Some hospitals opted to complete their current supply of old charts before adopting the new ones, while in some hospitals, the new charts were incorporated into the file and the old charts were left intact. Bound booklets were printed outside the hospital on a regular schedule, while for loose-leaf files, charts were either printed at the facility or by an external supplier. For instance, in one hospital that used bound booklets, the county administration had procured over a year’s supply of booklets, implying that removing old charts contained in such booklets would be wasteful. Nurses, therefore, had to find creative ways of appending the newly designed charts into the booklets- for example, using tape. Hospitals using loose-leaf files who printed charts on location found it easier to incorporate the new charts because they could assemble patient files on the go (illustrated in the quote below), while those that printed charts outside the facility found it tedious to undo the prepared file and incorporate new charts.

“*Even when we did away with the other two charts*, *the intensive and the standard one [old charts]*, *it was so*, *it was also very easy to transition from those ones to these ones because it is just a folder and a tool and you attach you make a file”*. *IDI 07 Nurse H3 [hospital with loose leaf-leaf files]*

The data clerks who are trained health records information officers played an important role in ensuring that the monitoring charts were available in the newborn ward by following up on printing with the records department, inserting the charts in the patient file and following up with the nurses whenever charts were available but unfilled. The nurse-in-charge also followed up with the records department to request additional charts to be printed and specify which charts they require in the newborn file. In one hospital, the nurses preferred to insert the charts in the patient file as needed, while in another, the staff insisted that the records department take on that responsibility as they had other duties to attend to as is highlighted in the following quote from a focus group discussion with data clerks.

“*Yes*, *in the beginning*, *I used to take [the charts] to the nurses*. *So*, *they never used to insert in the files*. *We went to intervene they told me they have no time for inserting extra papers in the file*. *They want the file that it’s complete*. *So*, *I had to take it to our Records Department and requested them if they can be inserting those forms in the files because it’s needed*. *So*, *at times they do it*, *at times they don’t*. *So*, *so sometimes when I have time*, *I normally go and insert them myself*.*” Data clerk from a hospital using loose-leaf files FGD R3 H12*

“*no*, *I wasn’t assigned but since it is one of my major tools*, *I take it as my responsibility to know*, *because if you don’t*, *then it means I’m going to miss a lot of things in my data*, *and that is upon me now*.*”Data clerk FGD R1 H11*

### Enablers of implementation

Factors that facilitated chart uptake were individual and team factors, complementary projects, and the removal of old charts. Chart uptake is defined here as putting the chart into the patient file and using it to document care.

The nurses-in-charge and paediatricians employed continuous training during ward rounds or during new staff orientation sessions to remind the junior staff such as medical officer interns and nurses to document on the chart. Additionally, they supervised documentation done by students to encourage chart uptake. Where the nurse-in-charge and paediatricians embraced the chart, they took the time to explain to their staff the importance of the chart and encouraged its use. They also contributed to ensuring that the charts were well-stocked by following up whenever there were printing challenges. Teamwork between cadres was also identified as an enabling factor of chart implementation, particularly because the chart is designed to be used by the nurses and doctors. Inasmuch as the unit leaders made effort to ensure the chart is adopted, some felt that ultimately, it all came down to individual motivation.

“*…actually we were doing it as a team when the round is on and we have the consultant and the Medical Officer if I find out that there was a problem somewhere I informed the Medical Officer[medical doctor without specialist training] that can you now add more energy to those people to tell them that we actually have to use this thing and they have to do their part and we do our part*. *The clinicians part*, *the nurses part*, *so if we do our part and their part remains*, *we really tell them you just have to do your part because we have roles here to play*.*” IDI 01 Nurse in charge H1*

“*Yeah*, *they have been supportive*, *they are always making sure that we have the tools so that when they are reinforced*, *we write all the values*, *we have where to write*, *we have the tools*. *And also*, *they have been very supportive” IDI 07 Nurse H3*

In hospitals where the NEST program was being implemented, staff received additional training on chart use which reinforced the initial training. In the newborn clinical audit study, the staff conducting the audits were required to extract information from the nursing notes to understand patient care. With time, it became clear that it was easier to extract this information from one chart rather than perusing notes in the entire file and therefore the newborn ward staff found the chart to be valuable as seen in the quote from the in-depth interview. Therefore, complementary projects within the CIN-N facilitated the uptake of charts in some hospitals.

“*We are five*, *five paediatricians*. *Initially*, *it was just me because I am the one who’d be involved and because am the one who had worked with you*, *then once people saw the importance of it and also through the audit again the other paediatricians saw the importance of it and started also enforcing*. *I mean some of them are also NEST trainers including two of them so now since they started working with the NEST program they’ve like now really taken up enforcing this documentation in the monitoring chart” IDI 03 Paediatrician H5 (clinical audits and NEST implementing hospital)*

“*you know in every training where we go to train staff working in newborn unit*, *as part of whatever content we are teaching we always have a monitoring section*. *And under the monitoring section*, *we have among other things the use of that chart*. *And it’s not like we are pushing it on people*, *but we feel it’s a chart that’s quite comprehensive that if used well it could make a difference and many of the facilities” IDI 06 NEST coordinator*

Lastly, where the old monitoring charts were completely removed, staff had no option but to use the new comprehensive monitoring chart. The early adopter hospitals (H11 and H3) had been involved in chart pilot at the request of the Nurse-in-charge or paediatrician between July 2019 and February 2020 and continued to use it beyond the piloting phase.

“*Yes*, *but in terms of usage of*, *okay*, *but in terms of the usage of comprehensive charts*, *we are coordinating well*, *it is being used very well*, *in fact*, *I had even started to forget the one we used to use in the past*.*” Data clerk FGD R4 H1*

“*We don’t have unless if you go to the old stationery*, *you can get them and in fact*, *they look weird when you compare it with the comprehensive one*. *They look weird*.*” IDI 07 Nurse H3(early adopter hospital)*

### Perceived benefits

All interview and FGD participants reported various benefits of the comprehensive monitoring chart in their work. Having consolidated information on one page was highly appreciated; with the chart being termed as a ‘one-stop-shop’; a design feature that was emphasized during the chart development phase. Subsequently, it made the health workers’ work easier by reducing the effort required to look for information and improving patient monitoring by making information available. Other benefits were that the chart was easy to use because less writing is required and there were fewer papers to document care.

“*Yes*, *I think a lot has changed because now it’s easy to document you know once you open that page you’re able to see what is not done*. *But you know initially*, *you were to open like four pages so if you don’t open the observation chart you’ll not see that they were not done or if you don’t open the feeding chart*. *But now even in the morning when you’re taking a report because we/ us nurses we hand over with the file so you read from the comprehensive chart*, *it’s easy to note in the morning what was not done and what was missed and the team are able to rectify rather than just giving you know handing over with everything like us we hand over with the file so you’ll use that comprehensive chart to hand over” IDI 04 Senior Nurse H4*

### Challenges and workarounds

Various challenges with chart adoption were reported relating to the staff and work environment (how work is organised), inadequate supply of charts, alternative places to document, and inadequate equipment.

Staffing and working environment challenges such as staff shortages, attrition, rotation, working in multiple departments (newborn and paediatric wards), and other competing tasks contributed to the staff experiencing a high workload. Additionally, during the early phases of the COVID-19 pandemic, medical and nursing students who typically relieve regular hospital staff of some duties were unavailable due to school closures, thereby aggravating staff shortages and workload.

From the interviews, it emerged that there were different shift patterns between nurses and clinicians in some hospitals. This meant that the feed and fluid prescription would be filled after the nursing shift had begun and likely documented in the doctor’s notes rather than the monitoring chart, leading to the prescription section of the monitoring chart being unfilled. The paediatricians, reflecting on chart uptake among medical and clinical officers, felt that they, as leaders needed to put in more effort in emphasizing that the feed and fluid prescription should be done on the comprehensive monitoring chart. The clinicians commonly worked in both newborn and paediatric wards, which meant that they were required to switch between writing the feed and fluid prescription in the doctor’s notes or treatment sheet in the paediatric ward and writing on the monitoring chart while in the newborn ward. Regarding supervising documentation and ensuring continuity of documentation, it was difficult to follow up on chart use at night when no paediatrician was on duty, while in other instances, chart uptake was interrupted when the senior nurse was away on leave or there were breaks in admission due to industrial action by nurses and doctors. In these situations, refresher training had to be done. Lastly, the staff reported having many other competing tasks which made it difficult to document as per recommended standards.

“*Due to the shortages*, *when you are alone you are not even able to document or implement the care required so when we have students the work is easier*, *and they easily also follow instructions*.*” IDI 02 Senior nurse H10*

Challenges such as printer breakdown or lack of printing paper resulted in a shortage of charts in some hospitals, which led to staff improvising charts or documenting care in other places. The availability of alternative places to document care also posed a challenge to using the comprehensive monitoring chart. For instance, the nursing Kardex used for documenting nursing care was considered an important job aid likened to a ‘Bible’ due to emphasis on documentation traced back to training institutions. In one hospital, the nurses viewed the Kardex as central to their practice and therefore felt the new monitoring chart was duplicative. Contrarily, in another hospital, the nursing Kardex was less used because the nurses found it easier to document on the new monitoring chart.

“*But the only*, *I think the only challenge is*, *is sometimes we normally have shortage[of charts]*, *and now when we get shortage*, *they try to draw*. *Sometimes they normally miss out some things” Data clerk FGD R2 H3*

“*I think is the same as the doctor’s notes*, *whereby they know*, *I’m supposed to write the continuation form*, *I’m supposed to write his admission form*, *so it’s just*, *it’s just in the mind*. *And then the training from the school*, *you know that nursing Kardex is like your Bible*. *We are taught Kardex is your Bible [laughs]*.*” IDI 10 Nurse-in-charge H9*

Chart users had to face the question of long-stay patients (e.g., premature babies) vis-à-vis how many charts would be sufficient to include in the patient file. Nurses and data clerks felt that for pre-term babies or babies needing kangaroo mother care, who require extended admission, the patient file will become bulky and difficult to handle. The question of long-stay patients was particularly a concern for those hospitals using bound booklets because the file was procured with a fixed number of charts, showing the complexity of using the charts in the existing system.

“*Yes*, *like if the baby*, *if the baby does not have a long length of stay*, *then that paper / that means the comprehensive chart will still be neat*. *But if it’s a bulky file*, *you have to insert a lot maybe more than three comprehensive charts and then they become worn out*, *they fold*, *now it becomes so difficult for me to get information*. *Like not difficult but I’ll say it becomes*, *it’s not easy to access the*, *to access the information because they have folds on them*.*” Data clerk FGD R5 H9 [hospital with bound booklet files]*

The following quote illustrates a preference for the old chart for long-stay patients because it could be used over a longer period as opposed to the comprehensive monitoring chart which is used over 2 days.

“*Yes*, *it [old charts] still exists*, *because this other one*, *they only use it for 48 hours*. *Since we have printing issues*, *you can’t put it because you find even a child stays more than 20 days*. *So*, *you have to insert around even ten*, *in the file*.*” Data clerk FGD R3 H12*

Lastly, drawing from the interview data, it was apparent that where relevant equipment was not available or not working, it was difficult to document the corresponding readings on the monitoring chart. For example, when the thermometer or pulse oximeter was not working, the temperature and oxygen saturation readings were left undocumented. Similarly, the lack of syringe pumps made it difficult to document the volumes of intravenous fluids given and calculate deficits accurately. Some hospitals reported using volumetric solusets that were inappropriate for the newborn population which in turn led to babies receiving too little or too much fluid.

“*We*, *we use the volumetric sets to infuse our fluids because we don’t have the pumps*. *So*, *usually two [portions of total volume prescribed] so that to prevent the overrun*, *that can happen when the baby changes the position of the hand because when the baby changes the position of the hand you find that you just hanged the fluid the other minute and it’s over”*. *IDI 07 Nurse H3*

“*Okay*, *with filling of the charts*, *the ones they normally fill they try*. *Its*, *it’s only that they have been complaining of SPO2 machines*. *They told me they have four and but only one is working but not even well*. *So*, *when they had a meeting this week*, *the consultant said that they are working on it to see that they*, *the SPO2 machine are working well*.*” Data clerk FGD R3 H12*

Some newborn ward staff reported various workarounds to manage the high workload challenge. From the interviews, two hospitals reported practising family-centred care which allowed staff more time to attend to sicker babies. They involved parents by teaching them how to care for their babies and to recognise danger signs, appropriate feeding, breastfeeding practices, as well as how to document feeds. However, this was not without challenges as questions were raised about whose responsibility it is to document tasks executed by another person, considering that the medical record is a legal document. In another instance of workarounds, nurses enlisted some mothers to provide peer-to-peer support to fellow mothers including documenting care. This idea faced challenges and was abandoned because of a lack of appropriate supporting policies. Lastly, there was a suggestion to implement advanced equipment that would make it easier to collect multiple vital signs as opposed to one vital sign at a time was needed, allowing the nurse to attend to other tasks.

“*We are because initially*, *we were making them involved without putting down the records*, *but we realized*, *you don’t have time to do it to compile that report for everyone by the end of the shift so the documentation was suffering at the same time but now most of these moms are class eight form four leavers they are people who can do the charting*.*” IDI 04 Nurse-in-charge H4*

“*R*: *We were told we are not doing our work and that is not the patient’s work so it’ll all go back to the policies of the hospital and what you agree on*

*I*: *So*, *if that can be agreed on at a hospital level*, *do you believe it can be enforced*?

*R*: *Yes*, *they can help us*, *they are not sick a KMC mother is not sick when she’s stable and it’s not everybody*. *There are those who you can assess*, *you know not all mothers are enlightened so you can check those who can*. *There are mothers who can’t even write anyway it’s not everybody*. *So you’ll just have to assess who can write who cannot*, *who can do this*, *and they can help anyway” IDI 05 Nurse-in-charge H*2

### Recommendations for improvement

At the data collection 8 months after the virtual launch, we asked interview respondents and data clerks how the design and future implementation process could be strengthened.

The chart was designed to be used over two days, but in two hospitals, the nurses and one data clerk reported that it was being used over a longer period. To illustrate, if there were blank columns on the right-hand side, the health worker would add a new date at the top and continue documenting care until the sheet was filled up before turning over to the next page. Reasons for this were the desire to reduce waste and high workload—as the nurses are unable to monitor all patients at 3-hourly intervals. This led to suggestions to modify the chart design to cover more days. Other suggestions for improvement included adding sections to monitor blood transfusion, weight, oxygen, urine volumes and revising the feed and fluid monitoring section. Lastly, health workers felt monitoring could be strengthened if they were supported to identify out-of-range vital sign values and give clear actions to be taken. It was suggested that the information sheet provided together with the monitoring chart could be printed at the back of the chart or printed and placed in prominent places in the ward, so it is always available. Throughout the chart design, we received informal requests to improve the treatment charts and the newborn ICU chart for the tertiary hospital.

“*So*, *I thought one day*, *maybe it needs to be combined*, *maybe is it A4 but with another one*, *like you can fold it and look*, *and have a space where what intervention was done at that point*, *time and the intervention done*, *something like that*. *Because you can’t fill up this with intervention*, *it has to be a document attached to this chart*.*” IDI 09 Paediatrician H6*

To strengthen uptake, respondents suggested that future implementation should be accompanied by mentorship or close follow-up, peer experience sharing, training at the hospital and in pre-service institutions as well as wider stakeholder engagement. They also suggested that feedback on documentation quality facilitated by documentation audits be included as part of the implementation process. Additionally, it was suggested that incorporating the chart into existing training programs such as NEST and the Emergency Triage Assessment and Treatment plus Admission course (ETAT+) would be instrumental in reinforcing the use of the chart.

“*I think*, *the way the tool was introduced in our unit*, *if you are planning maybe to*, *take the tool to another place that has never seen the tool*, *it would be important especially to first of all just to have a good CME with them so that they get to know that the tool has worked in other places*, *maybe get some testimonies from other places*.*” IDI 07 Nurse H3*

Lastly, there was a suggestion to improve the patient file by specifying the charts that need to be included and the order in which they should appear.

“*But having things mixed up in a filing system always messes up even the rounds become very difficult to do*. *People cannot track down what they need to get*, *and it becomes very problematic*. *But it’s something that maybe someone may need to figure out how to do it*, *and standardize that type of*, *of approach in terms of filing or keeping documentation for patients*.*” IDI 09 Neonatologist H6*

## Discussion

To improve the documentation of inpatient newborn care, we designed and implemented a comprehensive newborn monitoring chart in CIN-N. The nurses and paediatricians implemented the charts in their hospitals’ newborn wards following various procedures mimicking how a moderate scale-up of an intervention might unfold in a pandemic setting. We uncovered some contextual issues and elicited the perceptions of health workers on the chart as well as on the implementation process. Lastly, we elicited the users’ views on how to strengthen the process for a wider scale-up.

Although the hospitals in CIN-N are classified as county referral hospitals, their contexts varied as evidenced by the different filing systems, roles and chart implementation strategies. The type of filing system and availability of old charts that are printed periodically determined how quickly or how the newborn units added the new charts into the patient file. The nurses-in-charge played a role in ensuring monitoring charts are available by following up on supply, and in some cases, assembling the patient file, adding to their busy schedules. In contrast, nurses in other hospitals expected the health records department to provide a complete patient file. To train their staff, the nurses-in-charge used strategies such as continuous medical education sessions with all staff or one-on-one training to introduce the chart to other staff in the ward. These illustrations reveal how context can affect how an innovation is taken up. In their review, Palmer et al suggest that modifications to the setting is helpful to implementing innovations [20]. Our findings present an opportunity to modify the setting by clarifying roles concerning assembling the patient files to ensure seamless service provision. Therefore, a contextually informed implementation plan as part of a facilitated quality improvement process would be of great benefit for hospitals seeking to implement the chart or similar innovations in their setting. Similarly, in their review, Greenhalgh and colleagues suggest that technology is likely to be assimilated more easily if it is implemented with additional support, such as customisation and training [8].

To facilitate implementation, we engaged the nurse-in-charge and paediatricians who lead the newborn units to implement the monitoring charts at the hospitals. We anticipated that the nurse-in-charge who was our key contact person would be the “champion” thereby working with others in the unit to train and implement the chart. Champions are individuals who actively promote an implementation process and come from within the context where the implementation is taking place or external [21]. Likewise, internal, and external champions emerged in this study highlighting the diversity of roles as relates to patient medical records. The data clerks (external champions) were instrumental in our study to ensure that the new charts are printed and inserted into the patient file and occasionally followed up with the nurses whenever the charts were not used. They took up this role as the charts made their data extraction work easier but also because together with the health records department, were viewed as key liaison persons between the research team and the newborn units as concerns data collection. Additionally, complementary projects such as the NEST program and the clinical audits projects promoted the chart in two ways. The NEST program conducted newborn care training and emphasised the importance of documenting monitoring care through illustrations done on the chart. On the other hand, the clinical audit project encouraged the use of the monitoring chart as a source document for audits and emphasized ease of information retrieval whenever the new chart was used.

Stakeholder engagement is an important aspect of implementation research to support sustainable health interventions [22]. We engaged the nurses-in-charge and paediatricians as focal persons in the design and implementation phase. Members of the research team periodically contacted focal persons to provide support and understand the status of implementation progress. However, our engagement was not without challenges. The staff reported having many competing tasks in their line of duty and other quality improvement projects. Additionally, whenever a focal person was away (e.g., on leave), or was transferred out of the unit, the information on the new monitoring chart was not passed on to the incoming staff which negatively affected chart uptake. Further, due to COVID-19 restrictions on gatherings and travel, our engagement was done remotely which may have negatively affected the implementation but possibly reduced the hawthorn effect, thereby giving a clearer picture of adoption. The COVID-19 pandemic may also have affected health workers’ wellbeing with reports showing that there was limited information and a need for psychosocial support [23]. Competing priorities and staff turnover have been highlighted as challenges of engaging stakeholders [24]. These examples highlight the challenge of keeping stakeholders sufficiently engaged without overburdening them with additional meetings thus prompting the research team to consider different ways of engaging the staff at the hospitals to ensure that evidence is translated into practice. One opportunity that remains to be fully explored is the engagement of clinical champions to offer sustained support. Under the NEST program, each site has mentors that support the newborn care teams whenever they experience challenges. The mentors also participate in clinical audits and teaching during CMEs. They present a unique resource that can support documentation as part of wider quality improvement efforts. These champions can work closely with the records department to facilitate chart supply and with the health workers to ensure documentation targets are attained.

Working primarily with the nurses as the key focal person for this work may have inadvertently side-lined the clinical team and nutritionists thereby leading to poorer adoption outcomes for the prescription section that is filled by the clinicians and therefore partial adoption of the chart. To confound this, the medical and clinical officer interns who are likely to write a feed and fluid prescription were required to prescribe in a different place from where they have traditionally documented. They also served the paediatric ward that did not have similar monitoring charts and were reported to spend limited time in the newborn ward due to rotations that were required as part of their training. To facilitate seamless transitions and documentation between related departments, it would be beneficial to have similar monitoring charts in the paediatric and newborn wards thereby adopting a health systems approach [25] to improving documentation.

Challenges to the adoption of the charts included the availability of alternate places to document care, high workload and inadequate equipment. We encouraged the focal persons to remove the old charts, but this was largely dependent on the filing system in use, how much old stock was already printed and the support of the nurse-in-charge or paediatrician. In some facilities, the nursing Kardex was preferred for documenting nursing care even when the monitoring chart was available with one participant regarding it as a ‘Bible’. In this instance, the Kardex was viewed as an artefact that is tightly linked to nursing identity that they were reluctant to let go of. The presence of multiple documentation charts that collect similar or related information remains a challenge and provides an opportunity to define a newborn patient file by specifying which charts need to be included and in what order. Studies have investigated the duplication of data in paper-based medical records as part of efforts to streamline the documentation process, reduce documentation burden and prepare for electronic systems [26–28]. For this to be realised, a wider stakeholder engagement that includes the Ministry of Health and other relevant partners is required as well as addressing other issues such as staffing and availability of appropriate equipment.

We aimed to improve documentation of inpatient newborn care by designing and implementing a paper-based newborn monitoring chart using a Human-centred design (HCD) approach. During the design phase, together with workshop participants, we discussed feasible ideas given the context versus aspirational ideas. We received more suggestions from health workers (piloting and implementation phase) to improve the chart by adding items that were deemed important to document though these suggestions to add more items to the chart conflict with the common challenge of not having the time to fill the existing information. Additionally, for long-stay patients, the patient file might become bulky because it contains many charts. These suggestions illustrate the tension that arose between wanting the chart to do more but at the same time be simpler and require less documentation. Similar tensions are discussed by Steen where he argues that HCD practitioners need to balance understanding context and users’ needs versus applying new solutions [29]. The tensions and suggestions present an opportunity to explore other suitable solutions such as digital systems that may offer flexibility on how information is presented and embedded clinical decision support.

The implementation strategy involved a trainer of trainers model that was applied remotely in a network of hospitals. While this model was largely successful, there appear to be opportunities to strengthen the process. Results show that implementations might benefit from extended stakeholder engagement that is context adapted. The implementation innovations might also benefit from multiple use cases that facilitate its implementation–for example, the chart was used in the complementary projects which in turn played the role of facilitated implementation. On the other hand, if one is implementing an innovation as a stand-alone project, they might consider extended engagement with champions and a phased withdrawal from implementers. If the project spans more than one site, experience sharing between sites should be incorporated.

### Limitations

This study was conducted eight months after the virtual launch of the monitoring charts. During this period, there were uncertainties brought about by the COVID19 pandemic which led to limited gatherings such as continuous medical education sessions, limited physical interaction between the research team and hospital teams as had been the tradition and anxiety amongst health workers. The physical interactive sessions would have played a key role in facilitating sensitisation sessions with the newborn unit teams and providing opportunities for peer-to-peer learning as suggested by the participants. Further, there were extended industrial strikes during that period which may have interrupted chart uptake. This implies that our results may not reflect a typical setting. However, the study provides insights on how one might adopt a flexible implementation plan and work with a network of hospitals and it also provides lessons on scale-up of implementations.

## Conclusions

We successfully implemented a comprehensive newborn monitoring chart in a network of hospitals in Kenya in response to a need to improve documentation of inpatient newborn care. The chart implementation was enabled by initial and continuous training, complementary projects, the removal of old charts and the support of nurses-in-charge and paediatricians. The participants reported enjoying the benefits of having consolidated information in one A4 chart, less writing and fewer papers to flip through. However, the implementation was met with challenges related to the staff and work environment, inadequate supply of charts and inadequate equipment and physical meeting restrictions due to COVID19. These challenges provide opportunities to strengthen medical records by clarifying roles across departments, improving the chart supply process, improving staff induction to facilitate the transfer of information and specifying a newborn patient file to support information generation and use. Lastly, the participants suggested that future implementations should include training, peer experience sharing and mentorship as part of a facilitated implementation process that should be contextually adapted. In the next phase of this work, we will conduct a quantitative evaluation to understand the uptake of charts. In this way, we will build a comprehensive picture of the chart design, implementation, and evaluation with a view to inform future scale up beyond the participating hospitals.

## Supporting information

S1 Text Comprehensive newborn monitoring chart. (PDF)

S2 Text Information sheet. (PDF)

S3 Text PLOS’ questionnaire on inclusivity in global research. (DOCX)

S1 Data Data codebook with coding frequencies. (DOCX)
