# Supplementary material for: Implementing a comprehensive newborn monitoring chart: Barriers, enablers, and opportunities
Source: PLOS Glob Public Health. 2022 Jul 25;2(7):e0000624. doi: 10.1371/journal.pgph.0000624 (PMC10021603; doi:10.1371/journal.pgph.0000624)
Supplement: S1 Text — (PDF) [file pgph.0000624.s001.pdf]

COMPREHENSIVE NEWBORN MONITORING CHART

Version 2.8

**Name**	**IP NO**	**Sex** M □ F □ Indeterminate □	**D.O.A**	**D.O.B**
**Date today**	**Diagnosis**			
**Birth Wt** gm	***Interventions:*** *CPAP* □ *Oxygen* □ *Phototherapy* □ *Blood tranfusion* □ *Exchange transfusion* □ *KMC* □			

Daily Clinician Feed and Fluid prescription

Day of Life | **Current Wt =** gm

Total feed + fluid =______ mls/kg/day = _______mls

Feed: BF ☐ EBM ☐ Term Formula ☐ Pre-Term Formula ☐

Route: Cup☐ NGT☐ OGT☐

Volume & Frequency = _________mls 3hrly ☐ 2hrly ☐

24hr Feed Volume = _________mls

IV Fluid & Additives	Vol (ml)	Duration

Other prescribing instructions

Clinician's name **Time:**

Daily IV Fluid Nursing plan

Start time:

Hourly rate= _________ mls (____ drops/min)

Planned vol = _________ mls in_______ hrs

Monitoring

	Monitoring Freq___hrs | Time												
Vitals	Temp (^0C)												
	Pulse (b/min)												
	Resp Rate (b/min)												
	Oxy Sat (%) or Cy^0 Cy^+												
Assessment	Resp Distress 0,+,+++												
	CPAP Pressure (cm H_2O)												
	FiO_2 (%)												
	Jaundice 0,+,+++												
	Apnoea Y/N												
	Blood Sugar (mmol/l)												
	Completed by (name)												
Feed	Breastfeeding sufficient Y/N												
	EBM vol given (ml)												
	Formula vol given (ml)												
Fluid	IV volume given (ml)												
	IV Line working Y/N												
Output	Vomit Y/N												
	Urine Y/N												
	Stool Y/N												
	Completed by (name)												

Morning shift notes
Category: A□ B□ C□

For this shift: Total feed_______ mls
Total fluid _______ mls
Total feed/fluid deficit_______ mls

Completed by (name)

Afternoon shift notes
Category: A□ B□ C□

For this shift: Total feed_______ mls
Total fluid _______ mls
Total feed/fluid deficit_______ mls

Completed by (name)

Night shift notes
Category: A□ B□ C□

For this shift: Total feed_______ mls | Total fluid _______ mls
Shift deficit_______ mls
Total feed+fluid input in 24hrs_______ mls

Completed by(name)

24hr deficit______ mls

Jaundice 0 none, +mild(face),+++severe(feet)

Tick the category of baby after assessment

Alerts : circle readings outside normal range with red pen and action

[HOSPITAL NAME]

COMPREHENSIVE NEWBORN MONITORING CHART

Version 2.8

**Name**	**IP NO**	**Sex** M □ F □ Indeterminate □	**D.O.A**	**D.O.B**
**Date today**	**Diagnosis**			
**Birth Wt** gm	***Interventions:*** *CPAP* □ *Oxygen* □ *Phototherapy* □ *Blood tranfusion* □ *Exchange transfusion* □ *KMC* □			

Daily Clinician Feed and Fluid prescription

**Day of Life**	**Current Wt =** gm

Total feed + fluid =______ mls/kg/day = _______mls

Feed: BF ☐ EBM ☐ Term Formula ☐ Pre-Term Formula ☐

Route: Cup☐ NGT☐ OGT☐

Volume & Frequency = _________mls 3hrly ☐ 2hrly ☐

24hr Feed Volume = _________mls

IV Fluid & Additives	Vol (ml)	Duration

Other prescribing instructions

Clinician's name **Time:**

Daily IV Fluid Nursing plan

Start time:

Hourly rate= _________ mls (____ drops/min)

Planned vol = _________ mls in_______ hrs

	Monitoring Freq___hrs | Time												
Vitals	Temp (^0C)												
	Pulse (b/min)												
	Resp Rate (b/min)												
	Oxy Sat (%) or Cy^0 Cy^+												
Assessment	Resp Distress 0,+,+++												
	CPAP Pressure (cm H_2O)												
	FiO_2 (%)												
	Jaundice 0,+,+++												
	Apnoea Y/N												
	Blood Sugar (mmol/l)												
	Completed by (name)												
Feed	Breastfeeding sufficient Y/N												
	EBM vol given (ml)												
	Formula vol given (ml)												
Fluid	IV volume given (ml)												
	IV Line working Y/N												
Output	Vomit Y/N												
	Urine Y/N												
	Stool Y/N												
	Completed by (name)												

Morning shift notes
Category: A□ B□ C□

For this shift: Total feed_______ mls
Total fluid _______ mls
Total feed/fluid deficit_______ mls
Completed by (name)

Afternoon shift notes
Category: A□ B□ C□

For this shift: Total feed_______ mls
Total fluid _______ mls
Total feed/fluid deficit_______ mls
Completed by (name)

Night shift notes
Category: A□ B□ C□

For this shift: Total feed_______ mls | Total fluid _______ mls
Shift deficit_______ mls
Total feed+fluid input in 24hrs_______ mls
Completed by(name)
24hr deficit______ mls

Jaundice 0 none, +mild(face),+++severe(feet)

Tick the category of baby after assessment

Alerts : circle readings outside normal range with red pen and action
