# Supplementary material for: Implementing a comprehensive newborn monitoring chart: Barriers, enablers, and opportunities
Source: PLOS Glob Public Health. 2022 Jul 25;2(7):e0000624. doi: 10.1371/journal.pgph.0000624 (PMC10021603; doi:10.1371/journal.pgph.0000624)
Supplement: S2 Text — (PDF) [file pgph.0000624.s002.pdf]

[HOSPITAL NAME] NEWBORN MONITORING INFORMATION SHEET

Level of concern	Colour
0	Green
++	Amber
+++	Red

References

1. Mulvihill M, EOE Neonatal ODN NATT Tool Working Group. Clinical Guideline: Neonatal Alert, Trigger & Track (NATT) tool. 2018.
2. WHO. Thermal Control of the Newborn: a practical guide

Vital signs	Value ranges				
Temperature ^{0}C	Less than 35.5	35.6- 36.4	36.5-37.5		Greater than 37.5
Pulse (beats per min)	Less than 80	80-99	100 - 160	161-180	Greater than 180
Respiratory rate	Less than 30	30 - 39	40-60	61 - 80	Greater than 80
Oxygen Saturation % *On Oxygen*	Less than 90%		90 – 95%		Greater than 95%
On room air	Less than 90%	90 – 94%	95 – 100%		
Blood sugar (mmol/l)	Less than 1.9	1.9-2.5	2.6 – 6.6	6.7-9.9	Greater than 10

Respiratory distress classification

0	+	+++
No respiratory distress	Flaring	Grunting<br>Head nodding<br>Lower chest wall indrawing

These criteria are a guide only to increase surveillance on infants of potential concern. Readings in yellow and red bands should be acted on based on hospital guidelines.
