# Supplementary material for: Implementing a comprehensive newborn monitoring chart: Barriers, enablers, and opportunities
Source: PLOS Glob Public Health. 2022 Jul 25;2(7):e0000624. doi: 10.1371/journal.pgph.0000624 (PMC10021603; doi:10.1371/journal.pgph.0000624)
Supplement: S1 Data — (DOCX) [file pgph.0000624.s004.docx]

S1 Data – Data Codebook with coding frequencies

| Name | Description | Files | References |
| --- | --- | --- | --- |
| Challenges and workarounds |  | 13 | 118 |
| Challenges of documenting clinical care | Mention of things that make it difficult to document care properly or as per recommended frequency | 13 | 85 |
| Different nurse and doctor timings affecting documentation | instances where the differences in work reporting times make it a challenge to have complete documentation | 6 | 11 |
| documenting care in other places | mention of care being documented in other places in the file rather than the monitoring chart | 8 | 18 |
| Documenting in the kardex | Documenting in the nursing notes instead of the monitoring chart | 4 | 10 |
| Interruptions of ward operations due to external factors | e.g., strikes causing any gains on improving documentation that had been made to be undone | 4 | 9 |
| Lack of equipment or inadequate equipment | Instances where equipment such as thermometers are missing will lead to the corresponding vital sign not being documented or inadequate equipment | 4 | 11 |
| Staff rotation or attrition | Instances where staff spend limited periods in the ward then must move to other departments/wards. Or staff leave the ward | 7 | 15 |
| Staff shortage | low provider to patient ratio [ Medical/Clinical Officers/nurses] | 11 | 21 |
| long stay patients and number of monitoring charts |  | 6 | 9 |
| Workload | Things health workers do to help them manage the workload and document care | 9 | 24 |
| Involving parents |  | 6 | 11 |
| Suggestions to manage workload using technology | Eg, suggestions of equipment that can capture all vitals at once | 2 | 2 |
| Train those in the lower-level units | To manage the number of babies being referred and also empowering others | 1 | 1 |
| Triaging babies | mention of different intensities of documentation based on the condition of the baby | 4 | 5 |
| Enablers of implementation | Mention of things that facilitated chart implementation | 13 | 80 |
| Continuous training |  | 10 | 15 |
| Embraced by unit leaders | Role of unit leaders or those in authority | 9 | 14 |
| External facilitator |  | 3 | 4 |
| Individual motivation |  | 6 | 12 |
| leaders’ involvement in design |  | 1 | 2 |
| Old charts not used | Even when old charts are present in the file(in cases of bound files printed in bulk) they are not used | 2 | 3 |
| Old charts removed |  | 5 | 8 |
| Orienting new staff on the chart |  | 1 | 1 |
| others |  | 1 | 1 |
| Supervising documentation |  | 6 | 12 |
| Teamwork |  | 4 | 8 |
| Filing system context and roles | Information about how charts are printed, how they reach the ward and who is responsible. Include any challenges around printing the charts. | 13 | 91 |
| Challenges with printing |  | 5 | 11 |
| Lack of printing paper |  | 1 | 3 |
| Printer challenges | Any challenges that make it impossible to use the printer such as breakdown or lack of printer toner | 4 | 6 |
| Charts are always available | mention of never lacking charts | 3 | 5 |
| Complete lack of charts and workarounds |  | 2 | 7 |
| Filing system | Description of the various filing systems at the facilities and suggestions around the patient file | 4 | 12 |
| Bound booklet |  | 2 | 4 |
| adding extra charts to file |  | 6 | 17 |
| change to booklet |  | 1 | 1 |
| Loose leaf filing |  | 2 | 5 |
| chart handling | Description of how charts are handled during the admission | 1 | 4 |
| Including monitoring charts | Mention of how they attach the monitoring chart to the bound booklet | 1 | 5 |
| How charts reach the ward |  | 2 | 6 |
| improving access or availability of charts |  | 1 | 1 |
| Printing process |  | 3 | 11 |
| Charts printed at hospital |  | 1 | 3 |
| Charts printed by an external supplier |  | 2 | 6 |
| Who follows up | Mention of who is involved in ensuring the charts are available in the ward | 9 | 37 |
| Health workers | mention of nurses or clinicians being involved in preparing the patient file or inserting charts into an existing file | 4 | 13 |
| Records department | Mention of someone from the records department involved in preparing files or printing charts. This includes data clerks being involved | 4 | 21 |
| Implementation |  | 13 | 82 |
| Implementation and initial perceptions of the chart | Descriptions of how the chart was implemented, who was involved and initial perceptions | 11 | 33 |
| continuous training |  | 4 | 6 |
| Initial perceptions |  | 8 | 11 |
| Embracing the chart |  | 4 | 5 |
| Too much work |  | 3 | 5 |
| Initial training |  | 8 | 10 |
| who was involved |  | 5 | 5 |
| Practical use of charts | Description of how the various sections of the charts are filled from | 13 | 49 |
| How charts are filled |  | 0 | 0 |
| Clinician sections | Areas filled by the paediatrician/Medical Officer/ Clinical Officer | 7 | 13 |
| Good filling | where there is mention of charts being filled well | 2 | 4 |
| Nursing sections | Sections typically filled by nurses | 7 | 20 |
| Intention to improve |  | 3 | 3 |
| Single chart used for more than 48hrs | Descriptions of where a single chart is used over many days as opposed to the intended 48 hours | 4 | 7 |
| Uses of the chart | Any mention of the different uses of the chart rather than simple documentation. What is the added value? | 13 | 38 |
| Clinical newborn audits |  | 2 | 3 |
| Communicating | communicating to team members | 2 | 3 |
| Documenting care |  | 2 | 2 |
| Educating mothers |  | 1 | 1 |
| Facilitate NEST training and clinical discussions |  | 3 | 8 |
| Handover or summary |  | 5 | 7 |
| Supporting clinical management |  | 8 | 14 |
| Information sheet |  | 3 | 4 |
| Perceived or experienced chart benefits | perceived/experienced value and benefits or experiences of using the new monitoring charts | 13 | 26 |
| Captures everything | Any mention of information being in one place | 11 | 15 |
| Easy to use the chart |  | 2 | 3 |
| Improved monitoring |  | 1 | 1 |
| Less papers |  | 3 | 3 |
| Makes work easy |  | 4 | 4 |
| Recommendations for improvement | Suggestions around future implementations to other facilities | 11 | 42 |
| Charts to cover more than 2 days | Discussion around allowing the chart to be used over more than 2 days | 2 | 2 |
| Feed and fluid prescription |  | 2 | 5 |
| Identify champions |  | 3 | 4 |
| Including more items | Suggestion to include additional items to the monitoring chart | 5 | 7 |
| Documenting blood on chart |  | 1 | 3 |
| oxygen monitoring |  | 1 | 1 |
| Urine monitoring |  | 1 | 1 |
| Weight monitoring |  | 2 | 2 |
| Incorporate feedback | Send feedback to users on uptake of charts | 1 | 1 |
| Incorporate the chart in official documentation | Liaise with Ministry of health to get the chart approved as an official document and assigned a number | 1 | 1 |
| mentorship or close follow up |  | 1 | 3 |
| peer experience sharing |  | 4 | 5 |
| Recommendations for improvement | Suggestions to include anything that is not comprehensively captured in the monitoring chart or recommendations on how to improve the chart | 4 | 8 |
| Training | Strengthen the current training process | 4 | 4 |
